# Contemporary Biomarker Strategies for Patients with Chest Pain

**DOI:** 10.31083/j.rcm2305157

**Published:** 2022-04-27

**Authors:** Stephen Boone, W. Frank Peacock

**Affiliations:** ^1^Department of Emergency Medicine, Baylor College of Medicine, 1 Baylor Plaza, Houston, TX 77030, USA

**Keywords:** troponin, high sensitivity troponin, chest pain, acute coronary syndrome, biomarkers

## Abstract

This review focuses on the strengths and limitations of conventional and high-sensitivity troponin in the evaluation of patients with suspected acute coronary syndromes. High-sensitivity troponin (hsTn) assays represent a significant innovation over prior generation troponin assays. Owing to superior analytical precision, hsTn permits more rapid “rule-in” and “rule-out” of myocardial infarction. Furthermore, hsTn assays, in properly implemented clinical pathways, permit a reduction in the portion of patients requiring extended observation and testing.

## 1. Introduction

Acute coronary syndrome (ACS) is defined by an acute supply-demand mismatch in coronary blood flow resulting in myocardial ischemia. This spectrum of disease includes ST elevation myocardial infarction (STEMI) and non-ST elevation acute coronary syndrome (NSTE-ACS). The latter group is typically subdivided into either non-STEMI or unstable angina, according to the presence, or absence, of cardiomyocyte necrosis evidenced by abnormal serum biomarkers [1].

The importance of timely and accurate diagnosis of ACS in patients with acute chest pain, or chest pain* equivalent*, requires little discussion. Chest pain is one of the most common reasons for ED visits and contributes to substantial expenditures and resource utilization for patients and healthcare systems alike. Although the majority of patients with chest pain in the ED will not ultimately have a diagnosis of ACS or other cardiothoracic emergency, the potential consequences of a missed diagnosis are dire.

While there are numerous etiologies of chest pain, both benign and life-threatening, the focus of this review is the biomarker-based diagnosis of ACS. At the center of this endeavor is the complex challenge of reliably discerning which patients with possible ischemic chest pain and non-diagnostic ECG findings will require further short-term testing or interventions. Underdiagnosis may lead to harm in the form of major adverse cardiovascular events (MACE; commonly defined as death, myocardial infarction, stroke, or revascularization); overdiagnosis may result in iatrogenic harm, unnecessary hospitalizations and interventions, stress and anxiety, and low-value expenditures.

For all but the lowest-risk patients with chest pain, the history and physical exam lack adequate sensitivity to rule out ACS. Therefore, the electrocardiogram and troponin measurement are essential diagnostic and risk-stratification tools. A variety of diagnostic algorithms which incorporate history, risk factors, ECG findings, and troponin have been widely validated and implemented in emergency departments across the globe. Initially, these tools were studied in conjunction with conventional “sensitive” troponins, however, there is now rapidly expanding evidence and clinical experience supporting high-sensitivity troponin in conjunction with these decision tools.

Although we cannot fully separate other biomarkers from the discussion of clinical strategies and risk scoring systems, in this review we will primarily focus on the strengths and limitations of cardiac troponin (Tn). Neither sufficiently sensitive nor specific for a diagnosis of ACS, creatine kinase (CK), CK-MB, and myoglobin are effectively relics of a bygone era and will not be discussed.

## 2. Cardiac Troponins

The cardiac troponin complex regulates muscle contraction via calcium-mediated interactions of actin and myosin. This protein complex consists of three regulatory proteins: Troponin C, Troponin T (TnT), and Troponin I (TnI). Of these subunits, TnT and TnI, are the most specific to cardiac myocytes and are released into circulation in the setting of myocardial injury. It is important to note, that while serum TnI and TnT elevations have high specificity for myocardial injury of any etiology, they are not specific for acute coronary occlusion. Elevated Tn may occur with ischemic coronary disease, noncoronary cardiac disease, or noncardiac etiologies of myocardial injury [2]. Abnormal Tn values must therefore be interpreted in the context of the entire clinical scenario. Lastly, while TnI is thought to be unique to cardiac muscle, TnT is expressed to some degree in skeletal muscle and may be detected in the circulation of some patients with skeletal muscle disease [3].

In patients with symptoms suggestive of acute ischemic coronary disease, TnI or TnT measurements are useful to either “rule in” or “rule out” myocardial infarction. Clinical decision protocols incorporating troponin results should then be used to categorize patients into low, intermediate, and high-risk categories to guide subsequent diagnostic studies, treatment and disposition.

## 3. Conventional vs High-Sensitivity Troponin Assays

The troponin molecule may be measured using either conventional troponin assays or newer “high-sensitivity” troponin (hsTn) assays. In the context of troponin assays, “high sensitivity” refers to the *analytical sensitivity* of the assay, as opposed to clinical or statistical sensitivity. This is a very important distinction, as this improvement in sensitivity does not equate to a sacrifice in specificity when properly implemented into clinical pathways. High-sensitivity assays are able to detect and quantify much lower values of Tn compared to conventional assays, with measurable concentrations reported in over 50% of healthy subjects [4]. Sex-specific 99th percentile upper reference limits (URLs) in hsTn have been consistently identified among healthy populations of men and women. These differences have resulted in approval of sex-specific clinical decision cutoffs for various hsTn assays. Although sex-specific cutoffs, with lower values for women, increase the number of women with abnormal biomarkers of myocardial injury, it is not clear that using different cut-offs leads to improvements in clinical outcomes [5]. Nevertheless, the use of sex-specific cut-offs is currently advised [4].

Although hsTn assays were first introduced into clinical practice over a decade ago in Europe, Asia, Australia, and other countries, FDA approval of a hsTn assay did not occur until 2017 [6]. While there has been broad uptake in US hospitals and emergency departments since that time, however, hsTn assays are not currently universally available in the US. In the most recent guidelines from the American College of Cardiology/American Heart Association (ACC/AHA), which notably included representation from the Society for Academic Emergency Medicine (SAEM), hsTn was recommended as the preferred assay for evaluation of chest pain in the ED [2]. The authors note the existence of a “wealth of evidence” for the superiority of these assays in multiple aspects of chest pain evaluation, which we will further explore in this review.

## 4. “Rule-Out” with Conventional Troponin

Numerous accelerated diagnostic pathways (ADPs) incorporating conventional troponins have been developed and validated in the emergency department (ED) setting. The primary purpose of an ADP is to identify patients, with presentations potentially suggestive of an ACS, who can then be safely discharged from the ED after an ACS diagnosis is reasonably excluded. ADPs utilize clinical data in combination with EKGs and troponins at various intervals to objectively risk-stratify patients and guide clinical decision making. In *low-risk* patients with symptoms of at least 3 hours duration, and serially negative conventional troponins at 0 and 3 hours, ADPs have excellent negative predictive value for short-term MACE. For instance, both the HEART pathway and EDACS risk assessment, in conjunction with 0 and 2–3 hour conventional troponins, have demonstrated negative predictive values for 30-day MACE ≥99% [7, 8, 9]. Similarly, high negative predictive values for 30-day MACE were found in low risk patients identified in the ADAPT and ASPECT trials, which used 0 and 2-hour conventional troponins in combination with the TIMI (Thrombolysis in Myocardial Infarction) score, although the absolute number of discharge candidates was ~10% [10, 11]. In terms of permitting safe discharge and potentially reducing the need for downstream testing in low-risk patients, strategies using conventional troponins are effective, but only applicable in a limited number of patients [12]. However, challenges arise in applying these strategies to “non-low risk” patients who make up a significant portion of the population for whom ADPs are applied.

In the HEART Pathway trial, less than one-third of the patients were identified as low risk [8]. The EDACS score classifies approximately half of patients as low-risk, albeit with a lower sensitivity than HEART score [13]. Although some data would suggest that non-low risk patients with negative serial conventional Tn and non-ischemic EKGs have a very low likelihood of short term life-threatening events [14, 15], clinical guidelines would suggest further observation and testing in this group owing to unacceptably high rates of MACE, primarily driven by subsequent revascularization.

## 5. “Rule-Out” with High-Sensitivity Troponin

High-sensitivity assays are recommended over conventional assays by both US and European guidelines owing to superior analytical precision and growing clinical evidence of effectiveness. In addition to superior negative predictive values, hsTn assays permit more rapid “rule-out” and “rule-in” of myocardial infarction. Many clinical decision pathways originally developed with conventional troponins have been modified to incorporate these newer assays.

Although clinical protocols which use hsTn assays will typically incorporate a chest pain risk score (e.g., HEART, EDACS, TIMI, etc.), “hsTn only” protocols also exist. According to AHA/ACC guidelines, the recommended time-intervals for repeat troponin measurements in patients with >3 hours of symptoms before presentation, are 1–3 hours for hsTn versus 3–6 hours for conventional Tn assays. No matter which assay or protocol is utilized, subsequent measurements beyond these timeframes may be reasonable according to the specific clinical scenario and provider judgement. Furthermore, in patients with symptoms that began at least 3 hours prior to sample collection, guidelines support the use of a single hsTn result below the assay’s limit of detection to reasonably exclude myocardial injury.

When considering a proposed definition of “low-risk patients” as those with a 30-day risk of MACE less than 1%, this group can only be consistently identified with conventional troponins if serial measurements are obtained and the results are combined with a validated chest pain risk score. Moreover, when using such a risk score, if a patient falls into a “non-low risk” category due to history, age, risk factors, etc., then the results of conventional troponin testing do not move patients into a lower risk category. In contrast, hsTn assays, may allow a patient to move from a pre-test “non-low risk” category to a post-test “low risk” category when using either serial measurements or a single measurement obtained at least 3 hours after symptom-onset. Among a cohort of over 22,000 ED patients evaluated for possible ACS, with an MI prevalence rate of 15%, hsTn alone classified over half the patients as low-risk with 30-day risk of subsequent MI or death of 0.2% [16].

## 6. FDA-Approved hsTn Assays and Analytical Differences

At the time of this writing, there is an FDA approved hsTnT assay (*Roche Diagnostics*; Roche TnT Gen 5 STAT) and multiple FDA approved hsTn-I assays (*Abbott Diagnostics, Siemens, Beckman Coulter*). It is critical to recognize that each assay has different analytical sensitivities and references ranges which prevent direct comparison of results from one assay to another. Karády* et al*. [17] measured hsTn concentrations, using three different assays, in 624 patients with suspected ACS from the ROMICAT I and II trials. Using a 0/2-hour algorithm developed for each assay, the authors found significant discordance between assays in risk classification (rule-in vs observe vs rule-out) which would potentially impact clinical decision making. Although each of these assays has excellent diagnostic performance characteristics, it is important to recognize that there are differing cut-offs for each individual assay attributable to differences in analytical sensitivity and to the reference populations from which these cut-offs were derived.

We have previously highlighted the excellent sensitivity and negative predictive value (NPV) of a single hsTn value below the assay’s limit of detection (LOD). However, a caveat for this strategy, specific to the United States, merits further discussion. The FDA does not currently permit the reporting of hsTn values below the assay’s limit of quantification (LOQ; concentration at which the assay’s coefficient of variation is <20%). This restriction was historically in place owing to concerns of analytical precision at concentrations near decision cutpoints (e.g., the URL).

While seemingly miniscule numerical differences between the LOD and LOQ, the *sensitivity* for MACE at this higher cut-off may suffer slightly. In a retrospective study of over 7000 patients performed in multiple Canadian ED’s, Mcrae* et al*. [18] quantified the sensitivity and NPV of very low concentrations of hsTnT (Roche) in patients with possible ACS. One-third of patients had a hsTnT less than 5 ng/L (LOD), while an additional 8.5% of patients had a concentration less than 6 ng/L (LOQ). The 7-day MACE sensitivity for hsTnT <6 ng/L was 96.6% vs a sensitivity of 97.4% for cut-off <5 ng/L. It is noteworthy to highlight that these small differences in sensitivity resulted in an absolute difference in MACE NPV of just 0.1% (99.5% vs 99.4%), solely attributable to four additional coronary revascularizations with no differences in 7-day AMI or death. The clinical significance of this modest reduction in sensitivity for MACE, primarily driven by changes in revascularization, with preservation of excellent NPV and sensitivity for AMI is debatable. However, owing to this perceived limitation, some experts have advised the addition of a validated risk score to an initial hsTnT measure below the LOQ [19, 20].

## 7. HsTn Clinical Decision Pathways

**Fig. 1.**
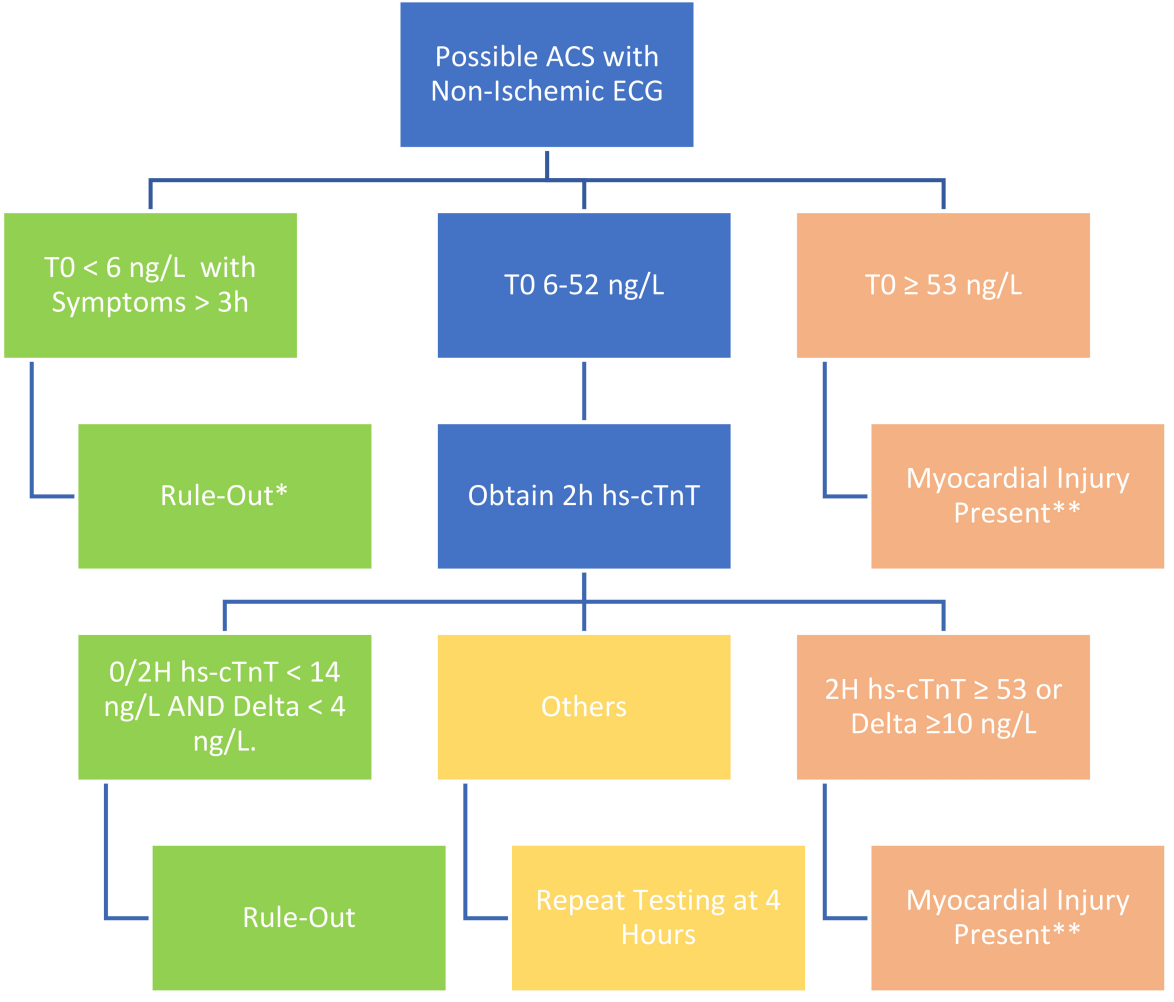
**Sample 0/2 hour pathway for rule-out of myocardial infarction with high sensitivity troponint (*Roche Elecsys*)**. *If symptoms less than 3 hrs, repeat troponin in 2 hrs. **If alternative explanation of myocardial injury or troponin elevation other than acute coronary syndrome (e.g., CHF, ESRD) consider repeat testing to assure stability. ACS, acute coronary syndrome; T0, time zero; Delta, change in troponin concentration over time.

**Fig. 2.**
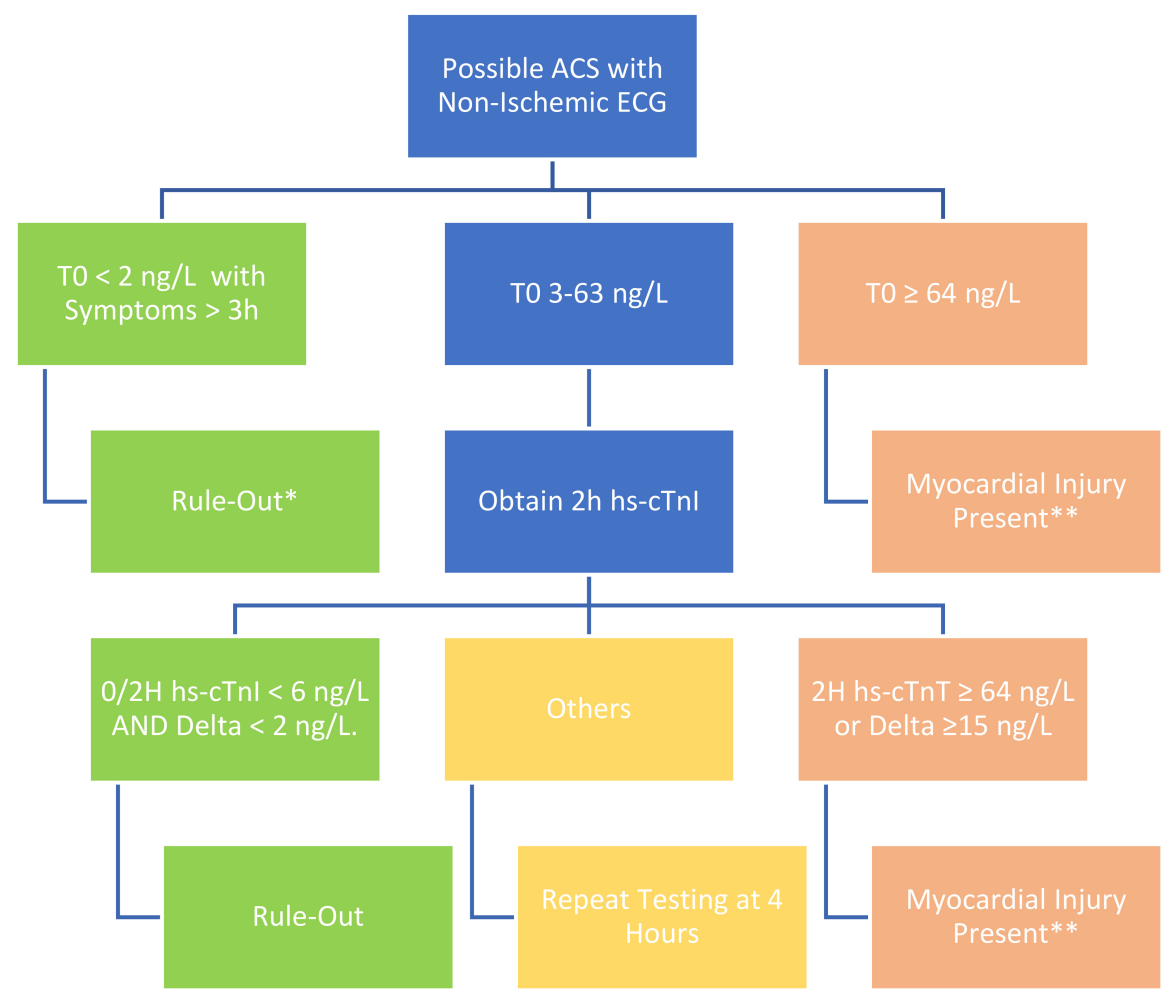
**Sample 0/2 hour pathway for rule-out of myocardial infarction with high sensitivity troponin I (*Abbott Architect*)**. *If symptoms less than 3 hrs, repeat in 2 hrs. **If alternative explanation of myocardial injury or troponin elevation other than acute coronary syndrome (e.g., CHF, ESRD) consider repeat testing to assure stability. ACS, acute coronary syndrome; T0, time zero; Delta, change in troponin concentration over time.

Rather than unstructured clinical assessments, institutions should implement agreed upon clinical decision pathways which include protocols for troponin sampling according to the particular assay in use. The pathways may include, but not necessarily require, inclusion of a cardiac risk score depending upon the particular hsTn assay and other factors [2]. To avoid confusion, it is advisable that institutions have a single hsTn assay available and not use both hsTn and conventional troponin assays [3]. Sample pathways for hsTnT and hsTnI are shown in Figs. 1,2. The most recent AHA/ACC guidelines also advise that the results of previous cardiac testing be considered when evaluating patients with chest pain in whom myocardial infarction has been excluded. The recommendations include considerations of the type, timing, and quality of the previous test results in addition to clinical factors such as changes in symptomatology [2].

## 8. Special Scenarios: False Positives, False Negatives

It is worth highlighting that troponin elevations in the setting of non-ACS cardiovascular disease should not be termed a “false positive”. Various conditions, such as heart failure, aortic stenosis, pulmonary embolism, and sepsis, among many others, may lead to troponin elevations. Although not necessarily indicative of acute MI, troponin elevations in these settings do represent myocardial injury and have important negative prognostic implications.

As mentioned, in a minority of patients with skeletal muscle disease, hsTnT may be elevated without obvious cardiac pathology; however, stable values on repeat testing in this scenario may be used to distinguish from the rising/falling values seen in AMI. Biotin supplements, most often taken in high doses for multiple sclerosis, and in lower doses for hair, skin and nail health, may interfere with hsTn assays which use biotinylation. Reports of falsely-low hsTn results prompted the FDA to release a safety communication to warn of biotin interference in troponin assays [21]. Although it appears this phenomenon is likely uncommon at biotin concentrations that would be expected with over-the-counter preparations [22, 23], providers should remain aware of this potential phenomenon. Hemolysis has also been reported to cause either falsely low or falsely elevated troponin values, depending upon the particular assay. Furthermore, interference may occur in the presence of heterophile antibodies, cardiac troponin autoantibodies, rheumatoid factor, lipemia, and hyperbilirubinemia [24]. Just as clinicians must take into consideration the entire clinical context (history, ECG findings, prior testing, etc.) when evaluating patients for a possible ACS, they must also consider factors such as sample quality (e.g., presence of hemolysis), host conditions, such as known or suspected circulating antibodies, and individual assay characteristics.

## 9. Special Scenarios: Chronic Kidney Disease (CKD)

HsTn concentrations above the 99% URL may be detected in over half of hospitalized patients with advanced CKD in the absence of AMI [25]. Elevated troponin levels in patients with CKD are likely due to a combination of reduced renal clearance of troponin and CKD-associated myocardial injury [26]. Although troponin concentrations are inversely correlated with the estimated glomerular filtration rate (eGFR), this relationship is non-linear [27]. This non-linear relationship, along with a high cardiovascular disease burden, poses challenges in the interpretation of elevated troponin in this population. Chuang* et al*. [25] in a separate review article for *Reviews in Cardiovascular Medicine*, provide an excellent summary of the literature on hsTn in CKD. In this review the authors highlight: (1) the higher baseline prevalence of MI in CKD, (2) an incremental reduction in specificity and positive predictive value of hsTn as eGFR declines, and (3) the preservation of excellent sensitivity and negative predictive value in accelerated rule-out protocols with hsTn in CKD. The ideal algorithm for management of an elevated hsTn in CKD is not well defined and likely too nuanced to explicitly capture in a simple flow diagram. Further studies are needed to determine if eGFR-adjusted references limits can be safely implemented into clinical practice. However, practically speaking, CKD patients without high-risk clinical findings and modestly elevated troponin should undergo serial troponin testing to differentiate chronic myocardial injury from acute ischemia. Patients with high-risk features and markedly elevated and/or rising troponins should be managed as ACS with early cardiology consultation. Similar to the approach in all patients with possible ACS, providers must weigh all relevant clinical factors, consider risks and benefits unique to the individual patient, and consider early cardiology involvement when the optimal path forward is not clear.

## 10. Conclusions

High-sensitivity troponin assays represent a significant innovation over prior generation assays. Experts in this field have mused that a better descriptor for these assays may be “high precision” troponins, as it is the precision and reproducibility at very low concentrations that truly sets these assays apart. These test characteristics permit both rapid myocardial infarction “rule-in” and “rule-out”. Furthermore, hsTn assays, in properly implemented clinical pathways, permit a reduction in the portion of patients requiring extended observation and testing. Although multiple reasonable pathways and protocols have been studied and validated, the optimal approach to chest pain evaluation continues to be refined. Institutions developing chest pain protocols should consider a variety of factors, including, but not limited to, unique assay characteristics, population disease prevalence, resource availability and access to follow-up care.

## References

[1] Thygesen K, Alpert JS, Jaffe AS, Chaitman BR, Bax JJ, Morrow DA (2018). Fourth Universal Definition of Myocardial Infarction (2018). *Circulation*.

[2] Gulati M, Levy PD, Mukherjee D, Amsterdam E, Bhatt DL, Birtcher KK (2021). 2021AHA/ACC/ASE/CHEST/SAEM/SCCT/SCMR Guideline for the Evaluation and Diagnosis of Chest Pain: A Report of the American College of Cardiology/American Heart Association Joint Committee on Clinical Practice Guidelines. *Circulation*.

[3] Wens SC, Schaaf GJ, Michels M, Kruijshaar ME, van Gestel TJ, In ‘t Groen S (2016). Elevated Plasma Cardiac Troponin T Levels Caused by Skeletal Muscle Damage in Pompe Disease. *Circulation: Cardiovascular Genetics*.

[4] Januzzi JL, Mahler SA, Christenson RH, Rymer J, Newby LK, Body R (2019). Recommendations for Institutions Transitioning to High-Sensitivity Troponin Testing: JACC Scientific Expert Panel. *Journal of the American College of Cardiology*.

[5] Bhatia PM, Daniels LB (2020). Highly Sensitive Cardiac Troponins: The Evidence behind Sex‐Specific Cutoffs. *Journal of the American Heart Association*.

[6] Chapman AR, Newby DE, Mills NL (2017). High-sensitivity cardiac troponin i assays in the diagnosis of acute myocardial infarction. *Heart Asia*.

[7] Stopyra J, Snavely AC, Hiestand B, Wells BJ, Lenoir KM, Herrington D (2020). Comparison of accelerated diagnostic pathways for acute chest pain risk stratification. *Heart*.

[8] Mahler SA, Riley RF, Hiestand BC, Russell GB, Hoekstra JW, Lefebvre CW (2015). The HEART Pathway randomized trial: identifying emergency department patients with acute chest pain for early discharge. *Circulation: Cardiovascular Quality and Outcomes*.

[9] Than M, Flaws D, Sanders S, Doust J, Glasziou P, Kline J (2014). Development and validation of the Emergency Department Assessment of Chest pain Score and 2 h accelerated diagnostic protocol. *Emergency Medicine Australasia*.

[10] Than M, Cullen L, Aldous S, Parsonage WA, Reid CM, Greenslade J (2012). 2-Hour Accelerated Diagnostic Protocol to Assess Patients with Chest Pain Symptoms Using Contemporary Troponins as the only Biomarker: the ADAPT trial. *Journal of the American College of Cardiology*.

[11] Than M, Cullen L, Reid CM, Lim SH, Aldous S, Ardagh MW (2011). A 2-h diagnostic protocol to assess patients with chest pain symptoms in the Asia-Pacific region (ASPECT): a prospective observational validation study. *Lancet*.

[12] Ashburn NP, Smith ZP, Hunter KJ, Hendley NW, Mahler SA, Hiestand BC (2021). The disutility of stress testing in low-risk HEART Pathway patients. *The American Journal of Emergency Medicine*.

[13] Boyle RSJ, Body R (2021). The Diagnostic Accuracy of the Emergency Department Assessment of Chest Pain (EDACS) Score: A Systematic Review and Meta-analysis. *Annals of Emergency Medicine*.

[14] Spiegel R, Sutherland M, Brown R, Honasoge A, Witting M (2021). Clinically relevant adverse cardiovascular events in intermediate heart score patients admitted to the hospital following a negative emergency department evaluation. *The American Journal of Emergency Medicine*.

[15] Weinstock MB, Weingart S, Orth F, VanFossen D, Kaide C, Anderson J (2015). Risk for Clinically Relevant Adverse Cardiac Events in Patients with Chest Pain at Hospital Admission. *JAMA Internal Medicine*.

[16] Neumann JT, Twerenbold R, Ojeda F, Sörensen NA, Chapman AR, Shah ASV (2019). Application of High-Sensitivity Troponin in Suspected Myocardial Infarction. *New England Journal of Medicine*.

[17] Karády J, Mayrhofer T, Ferencik M, Nagurney JT, Udelson JE, Kammerlander AA (2021). Discordance of High-Sensitivity Troponin Assays in Patients with Suspected Acute Coronary Syndromes. *Journal of the American College of Cardiology*.

[18] McRae AD, Innes G, Graham M, Lang E, Andruchow JE, Ji Y (2017). Undetectable Concentrations of a Food and Drug Administration-approved High-sensitivity Cardiac Troponin T Assay to Rule out Acute Myocardial Infarction at Emergency Department Arrival. *Academic Emergency Medicine*.

[19] Allen BR, Christenson RH, Cohen SA, Nowak R, Wilkerson RG, Mumma B (2021). Diagnostic Performance of High-Sensitivity Cardiac Troponin T Strategies and Clinical Variables in a Multisite Us Cohort. *Circulation*.

[20] Wu AHB, Kavsak PA, Aakre KM, Christenson RH, Greene DN, Apple FS (2020). Lot-to-lot Variation for Commercial High-Sensitivity Cardiac Troponin: can we Realistically Report down to the Assay’s Limit of Detection. *Clinical Chemistry*.

[21] U.S. Food and Drug Administration (FDA) safety communication (2019). Biotin Interference with Troponin Lab Tests - Assays Subject to Biotin Interference. https://www.fda.gov/medical-devices/in-vitro-diagnostics/biotin-interference-troponin-lab-tests-assays-subject-biotin-interference.

[22] Harley K, Bissonnette S, Inzitari R, Schulz K, Apple FS, Kavsak PA, Gunsolus IL (2021). Independent and combined effects of biotin and hemolysis on high-sensitivity cardiac troponin assays. *Clinical Chemistry and Laboratory Medicine*.

[23] Vroemen WHM, van Doorn WPTM, Kimenai DM, Wodzig WKWH, de Boer D, Bekers O, Meex SJR (2019). Biotin interference in high-sensitivity cardiac troponin T testing: a real-world evaluation in acute cardiac care. *Cardiovascular Research*.

[24] Januzzi JL, McCarthy CP (2020). Cardiac Troponin and the True False Positive. *JACC: Case Reports*.

[25] Chuang AM, Nguyen MT, Kung WM, Lehman S, Chew DP (2020). High-sensitivity troponin in chronic kidney disease: Considerations in myocardial infarction and beyond. *Reviews in Cardiovascular Medicine*.

[26] Chesnaye NC, Szummer K, Bárány P, Heimbürger O, Magin H, Almquist T (2019). Association Between Renal Function and Troponin T Over Time in Stable Chronic Kidney Disease Patients. *Journal of the American Heart Association*.

[27] Pfortmueller CA, Funk G, Marti G, Leichtle AB, Fiedler GM, Schwarz C (2013). Diagnostic Performance of High-Sensitive Troponin T in Patients with Renal Insufficiency. *The American Journal of Cardiology*.

